# Melatonin in the Treatment of Female Infertility: Update on Biological and Clinical Findings

**DOI:** 10.3390/biomedicines13102434

**Published:** 2025-10-06

**Authors:** Jan Tesarik, Raquel Mendoza Tesarik

**Affiliations:** MARGen Clinic, Molecular Assisted Reproduction and Genetics, 18002 Granada, Spain; mendozatesarik@gmail.com

**Keywords:** melatonin, antioxidant, immune modulator, female infertility, ovary, oocyte quality, uterine receptivity, placental function, endometriosis, adenomyosis

## Abstract

Many experimental and clinical studies published so far demonstrate that melatonin—produced mainly by the pineal gland located deep in the middle of the brain, between the two cerebral hemispheres, and in smaller quantities in a number of other organs and cells of the body—can be successfully used to treat different types of human female infertility. To accomplish this, melatonin acts mainly on the ovary, the uterus, and the preimplantation embryo, through its antioxidant, anti-inflammatory, and immunomodulatory effects, in addition to acting as a hormone and cytokine modulator. In particular, it increases oocyte developmental competence and uterine receptivity for the implanting embryo, improves placental health and function, prevents immune rejection of the implanting embryo and spontaneous pregnancy loss, and alleviates symptoms of endometriosis and adenomyosis. Yet, the use of melatonin in these contexts remains relatively limited, despite its convincing safety profile. This may be partly due to the fact that pertinent data concerning the use of melatonin in female infertility treatment are dispersed across various specialized scientific and medical journals, making it difficult for doctors and embryologists confronted with female infertility issues to access all of them. Therefore, this article was written to provide data that are easily understood. It draws from recent findings collected from different specialized journals focused on the molecular mechanisms of action, the clinical data, and the safety of this multifaceted biomolecule in the treatment of female infertility.

## 1. Introduction

Globally, one in eight women aged 16 to 74 has experienced infertility, defined by unsuccessful attempts at becoming pregnant over a period of a year or longer [1].

The treatment of female infertility has improved substantially over the last 80 years with the advent of assisted reproduction. The main milestones in this continuous process were marked by the discovery of ovarian stimulation [2,3], intrauterine insemination [4], ultrasound imaging of ovarian follicles [5], in vitro fertilization (IVF) [6], and the development of micromanipulation-assisted fertilization techniques [7,8]. Progressive improvements were also achieved in each of the above fields across time. However, in spite of these achievements, the success rates of assisted reproduction still remain relatively low [9].

The causes of female infertility may be associated with an impaired function of the ovaries (ovarian factor), the uterus (uterine factor), aberrant reaction of the immune system to the embryo (immunological factor), and a persistent chronic disease of the female genital tract, mainly endometriosis and adenomyosis [10,11]. Melatonin was first used to alleviate female infertility in the first decade of this century, when it was reported to improve pregnancy rates when used as an adjuvant in IVF treatment [12]. Subsequent review articles highlighted the possible role of melatonin in different issues of the female reproductive function and its potential use for human infertility treatment [13,14,15,16,17,18,19,20,21,22,23]. The present review is focused on the latest data, both from experimental animal research and human clinical studies, to explain how melatonin could be used as a biomedicine to improve human female fertility, while still acknowledging the pioneering articles published previously.

## 2. Different Facets of Melatonin Action

Melatonin is a multifaceted agent combining different molecular mechanisms of actions in cells and tissues. It is also a hormone acting at its complementary receptors in target cells in addition to being a direct antioxidant agent and an immunomodulator influencing various aspects of the immune system [24,25,26].

### 2.1. Melatonin as a Hormone

**Figure 1:**
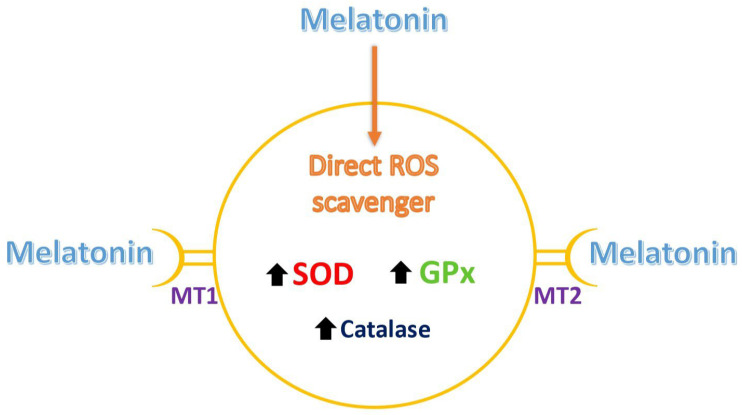
Alternative modes of melatonin action in target cells. Melatonin can act via its specific receptors (MT1 and MT2) to activate the production of antioxidant enzymes, such as superoxide dismutase (SOD), glutathione peroxidase (GPx) or catalase. In addition, as an amphiphilic molecule, it easily penetrates the cell membrane to act as a direct scavenger of reactive oxygen species (ROS).

Melatonin (N-acetyl-5-methoxytryptamine), first isolated in 1958 as a neuro-hormone mainly synthesized and secreted from the pineal gland, is now known to be secreted by a number of other cell types, including those of gastrointestinal tract, brain, eye, lungs, skin, kidney, liver, thyroid, thymus, pancreas, immune system and reproductive organs (reviewed by Fernando and Rombauts [12]). In mammals, melatonin acts at two types of receptors, MT1 and MT2, located at the cell membrane; it uses a G protein pathway for further signal transduction [23]. Since melatonin receptors are present in brain neurons, and its production is linked to the light/dark cycle, its most notorious effect is associated with the regulation of the circadian sleep–wake rhythm [24]. However, a number of studies have shown that melatonin can act through its receptors in a variety of cell types, producing various kinds of effects, in addition to acting as a direct antioxidant [25]. Hence, it is sometimes difficult to distinguish between the receptor-mediated and direct actions of melatonin in a particular condition (Figure 1).

### 2.2. Melatonin as a Direct Antioxidant

**Figure 2:**
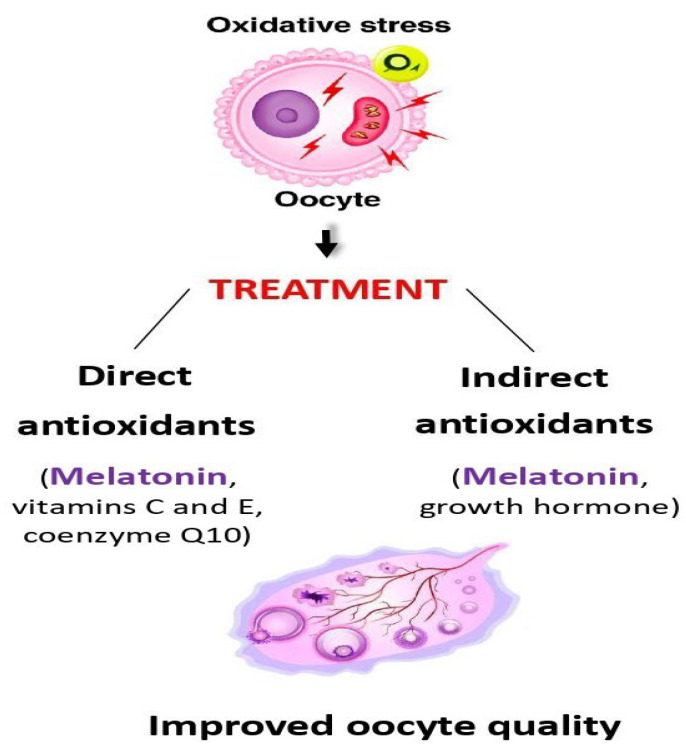
Treatment options to counteract harmful effects of oxidative stress on ovarian cells in general, and oocytes in particular. Melatonin can act as both a direct (receptor-independent) and indirect (receptor-mediated) antioxidant and can be combined advantageously with other types of antioxidants of both classes.

Owing to its amphiphilic nature, melatonin can easily penetrate into cells and exert effects independently of its receptor-mediated action. The antioxidant effect is one of its utmost receptor-independent actions. Unlike most other antioxidant agents, melatonin does not switch between oxidation-preventing and oxidation-supporting actions because, once the antioxidant effect is executed, melatonin does not come back to its original condition in which it could be both the receptor (antioxidant) and donor (pro-oxidant) of free oxygen radicals [26,27]. Consequently, melatonin does not alternate between the antioxidant and pro-oxidant actions, and it can also be employed as an antioxidant in combination with other molecules exerting the same effect (Figure 2).

Numerous studies have shown that mitochondria are both the main source of melatonin production and its target as a direct reactive oxygen species (ROS) scavenger (see Tan et al. [28] and Reiter et al. [29] for comprehensive reviews). In fact, melatonin does not penetrate into mitochondria by simple diffusion; rather, its entry is potentiated by a number of oligopeptide transporters, so that its intra-mitochondrial concentration is about 100-fold higher than in the plasma, as shown in mice [30]. This high melatonin intra-mitochondrial accumulation likely makes it one of the most potent direct ROS scavengers, thereby supporting mitochondrial function and protecting these organelles against the activation of pro-apoptotic pathways [28,29]. The improvement of mitochondrial health by melatonin—used alone or in combination with other antioxidants—is related to the reduction in oxidative stress. In addition to female infertility, this stress also contributes to a number of pathological conditions, including cancer, cardiovascular diseases, diabetes, neurological disorders (Alzheimer’s and Parkinson’s diseases, Down syndrome), psychiatric diseases (depression, schizophrenia, bipolar disorder), renal disease, lung disease (chronic pulmonary obstruction, lung cancer), and aging [31].

### 2.3. Melatonin as an Immunomodulator

In addition to its action as an antioxidant, melatonin also directly affects the immune system, making it more “intelligent” and less invasive towards healthy cells, as initially mentioned in 2022 [32]. In fact, as elucidated in ref. [32], melatonin attenuates the “primitive” innate immune response, destroying both affected cells and those still unaffected in their neighborhood, and favors a more “intelligent” means of action by reprogramming the activity of natural killer (NK) cells. This melatonin action is also important to mitigate symptoms of chronic diseases of the female genital tract (see below).

## 3. Melatonin Use in Different Types of Female Infertility

Melatonin was first used to treat human female infertility in the late 2000s (reviewed in Tamura et al. [33]), mainly to improve oocyte quality through its antioxidant action. Since then, in addition to its usefulness in the treatment of the ovarian factor, melatonin has also proven efficient in cases of the uterine factor, endometriosis and adenomyosis, and repeated implantation failure and pregnancy loss due to incompatibility between the immune cells within the uterine cavity and the antigenic status of the implanting embryo. Its usefulness also extends beyond the early embryonic development, encompassing obstetrics issues and lactation [23].

### 3.1. Ovarian Factor

#### 3.1.1. Ovarian Insufficiency

The original use of melatonin to treat the ovarian factor was directed to women with a low ovarian reserve. In fact, both age-related and premature ovarian insufficiency are associated with an increased production and/or decreased inactivation of ROS within the cells of the ovarian follicles [13,34,35,36,37,38] (Table 1) Both granulosa cells [39] and oocytes [40] express melatonin receptors (MT1 and MT2) on their surfaces, and the binding of melatonin to these receptors promotes the production of intracellular antioxidant enzymes, such as glutathione peroxidase, superoxide dismutase and catalase [33,41,42] (Figure 1).

In addition, because of its amphiphilic nature, melatonin penetrates easily into these cells, where it acts as a direct ROS scavenger [27]. Studies have shown that melatonin not only improves oocyte quantity and quality [13,34,35,36,37,38] but is also likely to slow down both age-related and premature ovarian decay [23]. The effect on age-related ovarian failure can be explained by the fact that intrinsic melatonin production decreases with advancing age [23], and its external administration—alone or in combination with other antioxidants—can restore normal melatonin levels in blood and follicular fluid (Figure 2).

However, it is also probable that pharmacological doses of melatonin can alleviate ovarian insufficiency unrelated to age, both a spontaneous one and that provoked by various pharmacological agents, such as chemotherapeutics. This latter idea was substantiated by a recent experimental study clearly demonstrating that, when used in combination with vitamin D3, melatonin mitigated premature ovarian failure induced by the chemotherapeutic agent cyclophosphamide in a rat model [47]. Another recent experimental study, performed in a mouse model, provided convincing evidence that melatonin alleviates premature ovarian failure by regulating granulosa cell autophagy through different molecular mechanisms [48].

#### 3.1.2. Polycystic Ovary Syndrome

Interestingly, in addition to its action against ovarian aging, melatonin was also reported to improve oocyte quality in young women with polycystic ovary syndrome (PCOS), as was reliably demonstrated in a randomized, controlled, double-blind trial involving five-hundred and twenty-six PCOS women [43]. The mechanism of this latter effect is less clear, but a clinical trial, performed with 32 normal male subjects, suggested that melatonin might stimulate the secretion of the growth hormone (GH) in humans, although the number of cases was too low to be completely convincing [49]. In fact, a higher-powered retrospective cross-over study clearly demonstrated a beneficial effect of GH administration on ICSI outcomes in PCOS women with a history of recurrent implantation failure [44] (Table 1).

A partial insight into the mechanism of melatonin effects on PCOS was provided by an experimental study performed in the rat model, which showed that melatonin was mainly effective via the MT1 receptor and suppressed the transport pathways of growth differentiation factor-9 (GDF9) to granulosa cells in antral follicles [50]. Another experimental animal study showed that PCOS mice treated with a combination of melatonin and metformin had lower testosterone levels and body weight, and more regular estrous cycles than those of the untreated PCOS group [51]. Whether melatonin acts in a similar way in women still remains to be determined. A recent study—combining an in vivo mouse PCOS model and in vitro analysis of human granulosa cells collected from PCOS and non-PCOS women undergoing in vitro fertilization—suggested that melatonin might improve ovarian mitochondrial dysfunction by regulating the circadian gene clock while also reducing granulosa cell pyroptosis in both of these systems [52].

### 3.2. Uterine Factor

The term uterine factor infertility is used for a wide range of conditions in which women fail to become pregnant because of some issue(s) related to their uterus, ranging from its complete absence to more or less subtle abnormalities of its structure and function. Understandably, this section only deals with those abnormalities that can be addressed by medical treatment, with a particular focus on fostering embryo implantation and post-implantation development. The term “uterine receptivity”, or “endometrial receptivity”, is traditionally used to denote the intricate processes undertaken by the internal lining of the uterine cavity (endometrium) and the uterine muscle (myometrium) to make the uterus receptive to the upcoming embryo entry [53].

Among a number of molecular pathways controlling uterine receptivity involving hormones, adhesion molecules, cytokines, and growth factors, many are potential targets for melatonin action. Experimental animal studies have shown that melatonin treatment enhances the process of implantation, reflected by a higher number of implantation sites, implantation rate, pregnancy rate and litter size compared to untreated females, as reviewed by Li et al. [23]. Regarding human data, the observation that melatonin supplementation improves uterine function has also been confirmed in women [54,55,56].

### 3.3. In Vitro Embryo Development Issues

During the time period between fertilization and implantation, newly formed embryos display an active metabolism generating a great amount of potentially harmful ROS. Under in vivo conditions, excess ROS are scavenged by intrinsic antioxidant enzymes present in the female genital tract. In contrast, when pre-implantation embryos are cultured in vitro, these natural antioxidants are absent and need to be substituted by culture medium supplements [22]. Owing to its dual mechanism of action (direct and receptor-mediated), melatonin appears to hold promise compared to other ROS scavengers under embryo in vitro culture conditions. In fact, independent in vitro studies with human embryos have demonstrated that melatonin improves the quality of human day-3 embryos in patients with a history of poor-quality embryo development. These studies also reported increased fertilization, cleavage, high-quality blastocyst development, implantation, and clinical pregnancy rates [22,55,56].

### 3.4. Recent Experimental Animal Studies

Recent studies with melatonin in animal models, including those with melatonin synthetic enzyme transgenic animals, have confirmed a significant improvement in oocyte and embryo quality as well as pregnancy rates and maternal milk quality [47,57,58,59,60,61,62].

### 3.5. Placental Health and Function

Melatonin exerts important beneficial effects on placental health and function [63] (see Li et al. [23] for a comprehensive review). In the human placenta, both the key melatonin synthases (AANAT and HIOMT) and melatonin receptors (MT1 and MT2) are produced by villous trophoblast from week 7 of pregnancy until parturition [64], with the highest expression being achieved during the third trimester of pregnancy [65]. Consequently, melatonin might modulate placental functions by alleviating oxidative stress (OS) through both receptor-mediated and direct actions, potentially depending on the stage of pregnancy [66]. In fact, placental OS is a condition known to be implicated in miscarriage and fetal growth restriction [67] and can lead to an inflammatory response causing preterm birth [68], and other inflammation-related pregnancy complications [23]. Prenatal melatonin supplementation alleviates placental hypercoagulability [69] and other fetal and perinatal sequelae caused by OS [23].

### 3.6. Gynecological Pathologies and Immunological Pregnancy Complications

Endometriosis and adenomyosis are the main gynecological pathologies that can be treated with success by melatonin. In spite of the distinct clinical manifestations, both of these pathologies share a common denominator: a failure of the intrinsic mechanism of apoptosis, also called programmed cell death (PCD), to help the organism remove the free endometrial cells detached at the end of the menstrual period by evacuating them through menstrual bleeding. In the case of endometriosis, these cells undergo a retrograde migration across the oviduct and settle on the surface of various intra-abdominal organs, mainly the ovaries and peritoneum, whereas in adenomyosis, these cells migrate into the uterine wall towards the myometrium.

**Figure 3:**
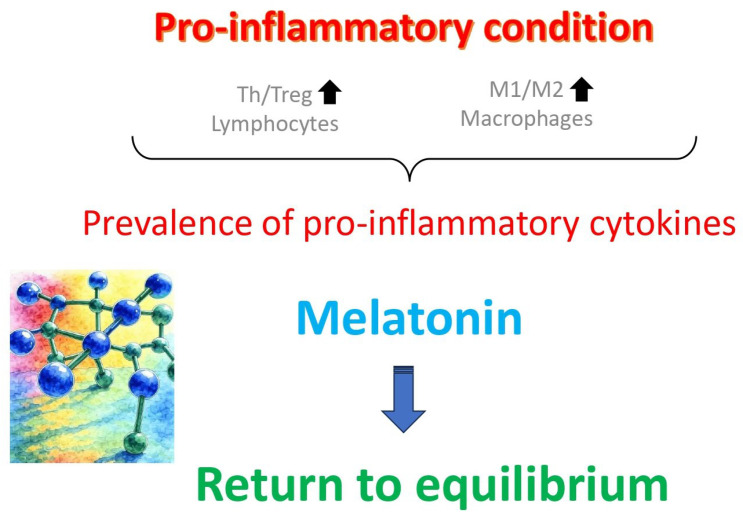
Several gynecological syndromes, such as endometriosis and adenomyosis, are caused by a dysregulation of the immune system. This is particularly marked by an increased prevalence of T helper (Th) lymphocytes over T regulatory (Treg) lymphocytes, along with an increased ratio of M1/M2 macrophages in the uterine cavity, with consequent prevalence of pro-inflammatory cytokines harmful for embryo implantation. Melatonin treatment reverts these anomalies and restores the normal uterine receptivity state.

The immunological mechanisms underlying both pathologies are similar and involve an imbalance between different types of immune cells (mainly lymphocytes and macrophages) and their products (Figure 3). This particularly includes increased T helper cell/T regulatory cell to pro-inflammatory (M1)/anti-inflammatory (M2) ratios, along with a prevalence of pro-inflammatory cytokines over anti-inflammatory ones [70]. In a controlled experimental study, melatonin was shown to revert this anomaly in adenomyosis mice, in addition to improving pregnancy outcomes [71].

One of the first animal experimental studies evaluating the effects of melatonin on endometriosis was published in 2013; it reported a regression of endometriotic lesions in rats and showed an improvement in their histopathological scores [72]. A more recent experimental study performed in the rat endometriosis model tested the efficacy of a combination of melatonin and colchicine and found that the simultaneous use of both medicines reduced adhesion of ectopic endometrial cells to the peritoneal surface by increasing apoptosis and decreasing survival of these ectopic cells [73].

In addition to these animal experimental data, melatonin was shown to revert this kind of immunological imbalance in vitro in humans as well, even though the exact molecular mechanisms of this effect still remain to be completely elucidated [46,74]. (Table 1). There are only few clinical studies dealing with the use of melatonin to alleviate endometriosis symptoms. Yet, in an RCT by Schwertner et al. [45], published as early as 2013, has demonstrated that oral melatonin (10 mg/day for a period of 8 weeks) reduced daily pain scores by 39.80% (95% confidence interval [CI] 12.88–43.01%) and dysmenorrhea by 38.01% (95% CI 15.96–49.15%).

The well-demonstrated safety of melatonin medication in humans, even at much higher doses than those sufficient to control endometriosis and adenomyosis [23,75], further supports its use as the first noninvasive causal therapeutic agent for endometriosis and adenomyosis [76]. In addition, this therapeutic action should be accompanied by other associated beneficial effects on female infertility [23,54] as well as preventive action against respiratory viral diseases [77] and certain forms of cancer [78].

## 4. Conclusions

This review provides convincing evidence in favor of the use of melatonin to treat human female infertility and to prevent a number of gynecological pathologies. By combining indirect (receptor-mediated) antioxidant action with a direct reactive oxygen species scavenger, melatonin improves oocyte and preimplantation embryo quality as well as the uterine receptivity for the implanting embryo. In addition, it modulates the immune system (mainly T lymphocytes and macrophages) to increase its specificity for pathogenic agents and lessen its destruction of uninfected cells. Through the same mechanisms, it relieves consequences of certain gynecological diseases, mainly endometriosis and adenomyosis. Taking into consideration its safety and lack of toxicity, melatonin is highly recommended as a first-line treatment in all types of female infertility, and as an adjuvant treatment in cases in which resorting to assisted reproduction techniques is required. Finally, more large-scale, robust clinical trials are still required to firmly establish melatonin efficacy and optimal dosing protocols across all recommended indications.

## Figures and Tables

**Table 1:** Summary of the studies using melatonin with human subjects in different clinical indications.

Study [Ref] (Type)	Indication	Delivery Route, Dose	Main Outcomes
Tamura et al. [34] (RCT)	Poor oocyte quality in ICSI	Oral, 3 mg/day	Improved fertilization rate
Eryilmaz et al. [35] (RCT)	Poor oocyte quality in ICSI	Oral, 3 mg/day	Increased mature oocytes and good embryos
Batioglu et al. [36] (RCT)	Poor oocyte quality in ICSI	Oral, 3 mg/day	Increased mature oocytes and good embryos
Nishihara et al. [37] (CT)	Poor oocyte quality in ICSI	Oral, 3 mg/day	Increased fertilization rate and good embryos
Jahromi et al. [38] (RCT)	Poor oocyte quality in ICSI	Oral, 3 mg/day	Increased mature oocytes and good embryos
Espino et al. [13] (RCT)	Poor oocyte quality in ICSI	Oral, 3 or 6 mg/day	Increased total oocytes and good embryos
Pacchiarotti et al. [43] (RCT)	PCOS	Oral, 3 mg/day	Enhanced oocyte and embryo quality
Vitale et al. [44] (RCOS)	PCOS and RIF	Oral, 6 mg/day	Improved IVF outcomes (pregnancy rate)
Schwertner et al. [45] (RCT)	Endometriosis	Oral, 10 mg/day	Reduced pain and dysmenorrhea
Qi et al. [46] (HBS)	Endometriosis	In vitro, 1 mmol/L	Inhibition of E2-induced invasiveness

Abbreviations: CT, controlled trial; HBS, hospital-based study; ICSI, in vitro fertilization by intracytoplasmic sperm injection; PCOS, polycystic ovary syndrome; RCOS, retrospective cross-over study; RCT, randomized controlled trial; Ref; reference; RIF, repeated implantation failure.

## Data Availability

Data are contained within the article.
